# The research examining factors associated with the opioid crisis among urban adults (REFOCUS): a mixed methods study protocol

**DOI:** 10.3389/fpubh.2025.1555214

**Published:** 2025-12-05

**Authors:** Mona K. M. Goggins, Paige Orphé, Jovonna Atkinson, Brittany Miller-Roenigk, Kaylee A. Palomino, Danelle Stevens-Watkins

**Affiliations:** 1 Department of Educational, School, and Counseling Psychology, University of Kentucky, Lexington, KY, United States; 2 Center on Drug and Alcohol Research, University of Kentucky, Lexington, KY, United States

**Keywords:** substance use, prescription opioids, opioid misuse, African American adults, non-medical prescription opioid use

## Abstract

**Introduction:**

Deaths due to opioid-related overdoses have increased across the United States. Specifically in the state of Kentucky, African Americans are the only population that continues to experience an increase in opioid-related deaths. Previous literature highlights African Americans having unique drug use patterns due to systematic barriers and how these barriers limit access to protective factors and substance use treatment. The current study aims to specify patterns of African American non-medical prescription opioid use (NMPOU) and how these patterns may differ based on biological sex and generational cohort. Also, this study plans on using this data on patterns to inform treatment modalities for African Americans.

**Methods:**

This study is a multi-aim, mixed-methods study that examines the patterns and factors of NMPOU and opioid misuse. Across the three aims, quantitative and qualitative data are collected to gain insight into the lived experiences of African Americans who use opioids, build upon public health knowledge of factors impacting African American opioid use, and outline substance use interventions for African Americans. Across the three aims, about 900 African American participants who self-identify as misusing opioids will be interviewed for data collection. Data analysis consists of power analyses to determine sample size, thematic analyses, and other quantitative data analyses to test the hypotheses in the study.

**Discussion:**

The REFOCUS study uses protective factors such as race-matching, experienced research assistants, and innovative recruitment strategies to attain and retain large sample sizes. Using these protective factors, the aims of the study are more likely to be successfully obtained.

## Introduction

Non-medical prescription opioid use (NMPOU) and opioid-related overdose mortalities have increased nationwide (1). Data suggests the highest increase in opioid-related overdose nationwide was among African Americans in 2020 (2). Research focusing on overdose historically relied on national datasets to examine rates. Through this method of data analysis, associated factors influencing opioid use for African Americans remain uninvestigated (3). By examining associated factors and patterns, a better understanding of how generational, biological sex, and other related factors increase risk for opioid use.

Previous research shows African Americans have unique drug use patterns that are influenced by structural and individual factors (4). Structurally, African Americans are more likely to experience incarceration and have a distrustful relationship with the legal system (5). These structural barriers are known to increase the risk of overdose in individuals reentering the community. These barriers also prevent African Americans from helping individuals who may have overdosed, even in areas with “Good Samaritan Laws,” or laws protecting those who report overdoses to authorities (6–10). On an individual level, factors such as peer and family drug use and perceptions of social support can also influence the NMPOU. These may differ based on biological sex and age (11–14). Outside of social influences, individual factors such as severe under-treatment of pain (15, 16), access to sources of illicit drugs (17), unfamiliarity with the value of specialized behavioral health services (18), and a lack of overdose education and naloxone availability (19, 20) impact the ways African Americans engage in NMPOU. These factors can also increase their risk for opioid-related overdose deaths.

In association with drug use patterns, African Americans take part in treatment-seeking behaviors differently than other groups. African Americans are less likely to complete treatment and receive evidence-based pharmaceutical treatments such as buprenorphine and methadone (21–24). Conversely, outpatient treatment options, such as self-help groups and outpatient rehabilitation centers, are utilized more by African Americans who engage in opioid use than by other groups (25). Research suggests these discrepancies exist due to general distrust of medications used to treat opioid use disorder, along with cultural barriers that prevent individuals from engaging in medicated treatment options (26). There is a clear need for research focused on risks associated with opioid-related overdose within African American communities. Findings from this study can inform treatment providers and community leaders poised to influence positive change and to better inform treatment utilization and opioid education for African Americans.

Kentucky remains in the top five states with the most opioid-related overdoses in the nation since 2020 (1). While statewide efforts have focused on reducing non-medical prescription opioid use and associated overdoses (27), African Americans in Kentucky are still experiencing an increase in overdose mortality (28). The present study seeks to address this by examining NMPOU among Black/African American adults in two urban counties. These two urban counties are home to a prominent African American community and have also been critically impacted. According to the 2022 Kentucky Overdose Fatality Report (28), of all the counties in the state, the largest county, which is included in the current study, reported the highest number of fentanyl-related drug overdose deaths with the second largest county, also in the current study, following in second (28).

## Objectives

This paper describes the Research Examining Factors Associated with The Opioid Crisis among Urban Adults (REFOCUS) Study. The REFOCUS study is a mixed-methods study on non-medical prescription opioid misuse among African American adult men and women between the ages of 18 and 69. This study seeks to investigate the generational and biological sex factors related to to increase in NMPOU in urban adults. The main goal of the study is accomplished across three aims with related goals. The goals of each aim and the associated factors examined are as follows:

- Aim 1: the study focused on examining biological sex and generational characteristics and how they are related to three patterns of drug use: NMPOU, other non-medical prescription drug use (NMPDU), and illicit drug use, as well as use of treatment services. This was done by recruiting 40 males and females across age cohorts and interviewing them about their experiences with drug use.
- Aim 2: the study seeks to identify complex patterns influencing NMPOU, other NMPDU, illicit drug use, and treatment service use among African Americans engaging in NMPOU. Factors such as family and peer relationships, social support, income, employment, health care access, active coping, and distrust in the healthcare system are examined in Aim 2. This is being done by recruiting 800 males and females across age cohorts and administering quantitative instruments about their experiences with substance use and other related factors.
- Aim 3: the study will assess findings from previous aims and their ability to inform substance use interventions by developing messaging for African American non-medical opioid users. Aim 3 messages will assess participants' attitudes toward factors of treatment. The anticipated result of this study is to produce informative literature on the biological sex, and generation-specific patterns in African American NMPOU. Further, this study anticipates using the results to increase treatment engagement, or the act of participating and completing treatment, and retention among African Americans who engage in NMPOU. This is being done by recruiting 40 males and females across age cohorts and interviewing them about their opinions on substance use and treatment engagement messaging.

The current study centered its data collection efforts around the Theory of Subcultural Evolution and Drug Use (29). This theory emphasizes understanding drug use trends through the lens of their social significance across different generations. The Theory of Subcultural Evolution and Drug Use has been applied to African American samples in previous literature to gain a clearer understanding of the generational factors that influence changes in drug use patterns, particularly related to substances such as crack, heroin, and marijuana (30–32). The REFOCUS study expanded on the literature related to this theory by examining the associated factors of NMPOU and how they may differ based on biological sex and generational cohort.

## Methods and analysis

### Study design

**Figure 1:**
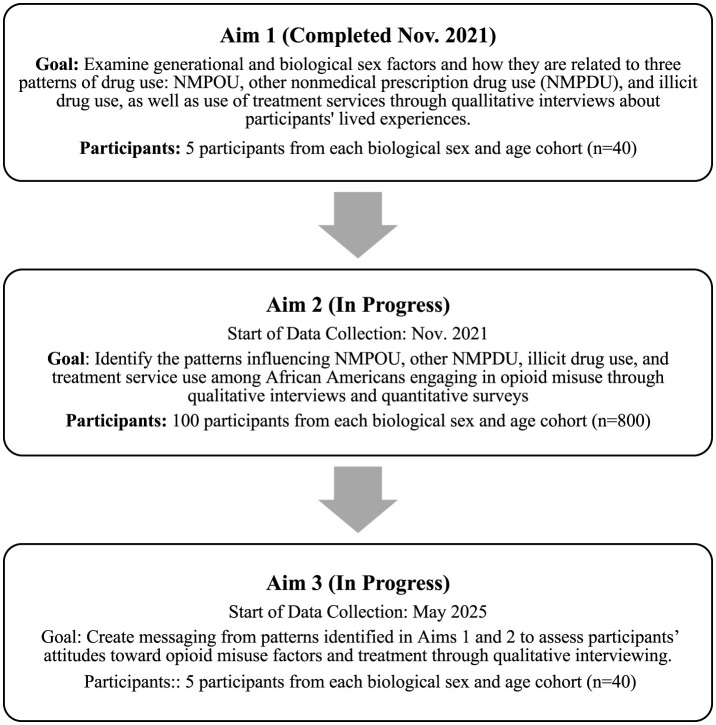
REFOCUS study timeline.

The REFOCUS study stratified participants by biological sex and age to examine the influences of non-medical opioid use among African Americans. Age cohorts consist of those born between 1955–1969, 1970–1979, 1980–1994, and 1995–2007. The following age cohorts are established based on the proposed drug subcultures associated with the Theory of Subcultural Evolution and Drug Use (29). These generational cohorts are each influenced by different aspects of drug epidemics. A mixed-methods approach is also used to facilitate comprehensive survey data on generational and biological sex factors influencing NMPOU and the use of treatment services, and to assess how various treatment methods increase treatment engagement among African Americans and decrease NMPOU. Neither patient nor public involvement was utilized when constructing the study's design, ethical considerations, or study outcomes. Aim 1 was completed in November 2021. Aim 2 started in November 2021 and is still in progress. Aim 3 began in May 2025, and data collection is in progress alongside Aim 2. A study timeline outlining each aim's goals and participant requirements can be found in Figure 1. Study design differences and data analysis plans for each aim are best described separately.

### Study recruitment

Recruitment methods for all three aims are identical. Flyers were posted at bus stops, parks, and other public areas in two urban counties in a southern state. The flyers included information about the project and instructed interested participants to call the toll-free study number or email the study contact. Flyers were also posted in local businesses in these areas (i.e., barbershops, libraries, convenience stores, etc.) with the permission of facility owners. Social media sites, such as Facebook and Instagram were used to promote the project. Digital versions of the flier including a description of the study and contact information were posted and shared on the social media accounts. All flyers included a QR code providing potential participants with more information on the study or directing them to the online screener to detect their eligibility for the study.

Media sources such as the radio and social media to reach the youngest cohort (1995–2006), which was the hardest cohort to recruit to the study. The 30-s radio ad was played on two local radio stations in an urban county Monday through Friday, during the week. These stations selected to play the radio ad are known to have younger, African American listeners, so this tactic helped the study recruit the youngest cohort. Social media presence was established by having research staff post research flyers on their personal social media pages to spread the word to different people. Later, the study created its social media page on Instagram where research flyers were also posted. Furthermore, the study's social media page aimed to build rapport with potential participants as it showed how else the study was involved in the Black communities it served. Pictures from resource events, staff biographies, and upcoming community events are posted to relate to the youngest cohort of the study and reach participants in a non-traditional way.

Recruitment also took a word-of-mouth approach during the second aim of the study to recruit more participants. This approach worked by calling past participants of Aim 1 to inform them they were eligible to take part in Aim 2 activities. Further, a participant referral system was implemented during Aim 2 of the study. The participant referral system allowed individuals who completed study activities to refer others who may be eligible for the study to earn additional compensation. Participants could earn $10 for referring a participant after they completed all study activities. The referral program allowed participants to refer up to three people, resulting in the potential for $30 additional compensation. Changes were made to the referral process during Aim 2 to improve enrollment, which increased the referral bonus to $30 for each participant, resulting in the potential to earn $90 in additional compensation.

The most crucial aspect of the study's recruitment strategies were the conections study staff made with the community and potential participants. These connections involved building rapport in the neighborhoods by providing food, toiletries, or other resources beneficial to the community and using basic rapport-building and listening skills (33). Research staff also encouraged those who may be eligible to fill out a screener in these conversations. To protect participant privacy, individuals interested in filling out a screener were removed from group settings to complete eligibility questions. Interviews were scheduled once the screener found the participant eligible to increase engagement in study activities. By creating connections in communities, REFOCUS became a trusted name, and potential participants of the REFOCUS study could connect with research staff more personally. This allowed REFOCUS to also partner with organizations in these communities.

### Participant sampling

The current study employed a purposeful sampling approach to achieve the study's goals. Study eligibility differed slightly based on the study's aim, and participants were eligible to complete study activities if they met the eligibility criteria at the time of recruitment. Specific eligibility requirements are included within each aim below.

#### Aim 1

##### Expected outcomes

In Aim 1, researchers expected to gain valuable insight into patterns of opioid use, treatment for substance use, and lived experiences of African Americans who use opioids through qualitative interviews. Different themes of opioid use, treatment participation, and experiences were expected within different age and biological sex cohorts. Themes related to characteristics associated with opioid use patterns were expanded upon during the data collection of Aim 2.

##### Participants

Aim 1 required 40 (*n* = 40) participants. Assessing the informational power, forty participants were enough to produce a saturated sample because the aim of the study was narrow, the criteria to participate in the study were specific, and the quality of qualitative data collection was rich and specific (34). Analyzing the study's data across cases and applying the study's theory after the data is collected requires more participants. Taking all factors into consideration when performing the study's informational power, each of the study's generational cohorts needed five biological males and five biological females to obtain the data needed. Participants of the study were eligible if they met the following criteria: (1) self-identify as Black and/or African American; (2) were between the ages of 18 and 65 at the time of screening (1955 was the birth year of those who were 65 in 2020 when the study began. Those who were born in this year could still participate, even if they are older than 65) as study years progressed; (3) were English-Speaking; and (4) had used a prescription opioid in a way that was not prescribed or differently than prescribed in the past 6 months. Participants consented to audio-recording at the time of their interview. Participants who completed Aim 1 could complete Aim 2 activities, but they cannot complete Aim 3 activities. Participants from Aim 1 cannot participate in Aim 3 activities because they are familiar with the study's aim to create messaging and interventions for urban communities. This may lead to biased responses from previous participants.

##### Data collection, incentive, and retention

**Figure 2:**
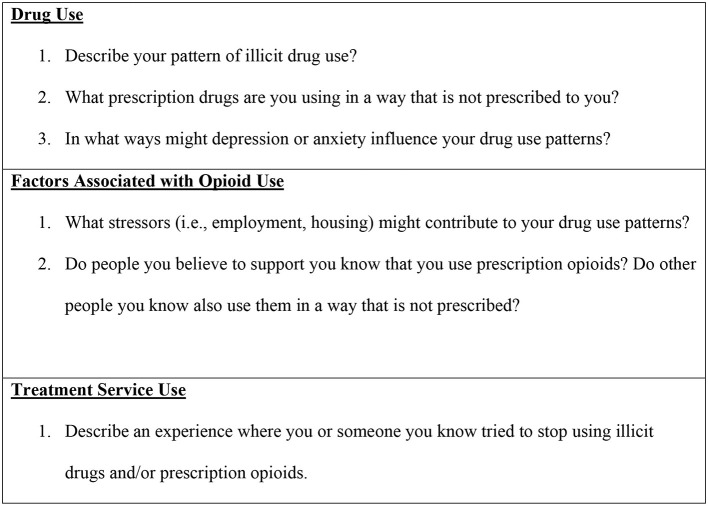
Sample questions: Aim 1.

Data from Aim 1 of the project was collected by African American students and fellows with a minimum of master's level clinical skills and a psychologist extensively trained in qualitative research methods involving African Americans in projects examining sensitive topics. Informed consent was obtained for the project's current activities and to provide permission for future contact involving other projects for which participants may be eligible. Participants were made aware they may decline project activities and leave the interview at any time. Face-to-face interviews at this stage of the study were conducted by age and biological sex cohorts. In-depth interviews continued until saturation for each biological sex/age group was reached (*n* = 5), resulting in 40 interviews total. Data saturation was achieved when all interviews were completed. Interviews lasted approximately 60–90 min and were audio-recorded and transcribed with integrated field notes of behavior observations. To protect against interviewer bias, research staff read each question to the participant in the same words as they appeared on the interview script. Also, a professional transcriber not involved in the research project transcribed the audio recordings. Participants were asked questions about patterns and antecedents of their drug use and their experiences with drug treatment. Examples of questions participants answered can be found in Figure 2.

For completing and participating in data collection activities, participants earned $40. If they were selected for member-checking and completed those activities, they received an additional $25. Data collected during a participant's interview would be de-identified and held in the study's database indefinitely. For Aim 1, participants were part of study activities for the duration of their interview time with the potential for random selection for a member check-in. For the member check-in, 16 participants, two from each age and biological sex cohort, participated in another 60-min session and were released from their participation.

##### Analytic plan

Aim 1 data was analyzed by a senior scientist and a graduate student, who are both trained in interpreting qualitative, using thematic analysis (35, 36). The six phases of this process are as follows:

1. Data was read multiple times to pull memos highlighting aspects of interviews that are relevant.
2. Data was coded and organized into meaningful groups based on its elements.
3. Coded data was re-read to connect codes into themes. A thematic map represented the relationships between the two.
4. Themes were re-evaluated to consider codes not yet fitting into the existing themes. Adjustments to the thematic map took place in this stage in this stage.
5. To ensure themes highlight participants' intended meaning and relate to the purpose of the study, two participants were chosen at random from each age and biological sex cohort (*n* = 16) to review if their experiences are accurately represented by the themes selected. An additional incentive was offered to these participants.
6. Recurring themes were reviewed with the entire research team and analyzed the key findings. These discussions helped finalize the questions used in Aim 2 of the study.

#### Aim 2

##### Expected outcomes and data hypotheses

Aim 2 study activities are currently in progress and expected to build upon drug use patterns and treatment service use found in Aim 1. Aim 2 outcomes are expected to provide data to create messages for Aim 3. Papers using this data are anticipated to reflect on NMPOU and its treatment services and how these patterns differ based on biological sex and generation. Two instuments are crucial in interpreting data from this aim. The Composite International Diagnostic Interview-Substance Abuse Module (CIDI-2-SAM) (37) is used to assess use quanity and frequency in participants along with other demographics about their substance use (i.e., age of first and last use, symptoms, and withdrawl symptoms). The Treatment Services Review (TSR-6) (38) is used in this aim to assess the types of treatment services utilized by participants in the study. When data collection ends for Aim 2, data analysis will also focus on testing hypotheses like the ones listed below:

- H1: Age of NMPOU initiation will be inversely related to the progression of use, as measured by the CIDI-2-SAM; biological males and biological females are expected to have stronger associations, but this relationship will be moderated by experiences in the criminal justice system.
- H2: NMPOU will be positively associated with physical health problems, mental health problems, and stress. These associations are anticipated in older biological males and biological females with access to healthcare.
- H3: Biological male opioid users will be less likely to initiate treatment services as measured by the TSR-6 when compared to biological females. The degree of distrust in the healthcare system will indirectly affect the relationship between biological sex on treatment (TSR-6).

##### Participants

Aim 2 requires many participants (*n* = 800), 100 biological males and 100 biological females from each age cohort. The minimum sample size was calculated by using a two-tailed level of significance (*p* ≤ 0.05). A minimum sample with at least 80% power to detect a small to medium effect size (*d* = 0.3) was detected at 90 participants per sex and generational cohort (i.e., 90 male participants in the 1955–1969 generational cohort, 90 female participants in the 1955–1969 generational cohort, etc.). To accurately account for the influence of biological sex and generational factors, and SEM path analysis (model fit by RMSEA) assumed seven measured variables, a sample size of 160 was needed to achieve 80% power for an acceptable model fit at alpha = 0.05. Therefore, 200 participants were enrolled needed for each generational group. At least 20 participants are needed for each parameter, therefore the sample size is considered large. Eligibility criteria are identical to Aim 1. Though, in the second aim, individuals are eligible for the study if they use illicit opioids in addition to using a prescribed opioid differently than prescribed to qualify for the study. Participants who complete Aim 2 could have completed Aim 1 activities, but they will not be eligible for Aim 3 activities. Similar to Aim 1, any Aim 2 participants may not complete Aim 3 study activities because of the increased possibility of biased responses from previous participants.

##### Data collection, incentive, and retention

**Figure 3:**
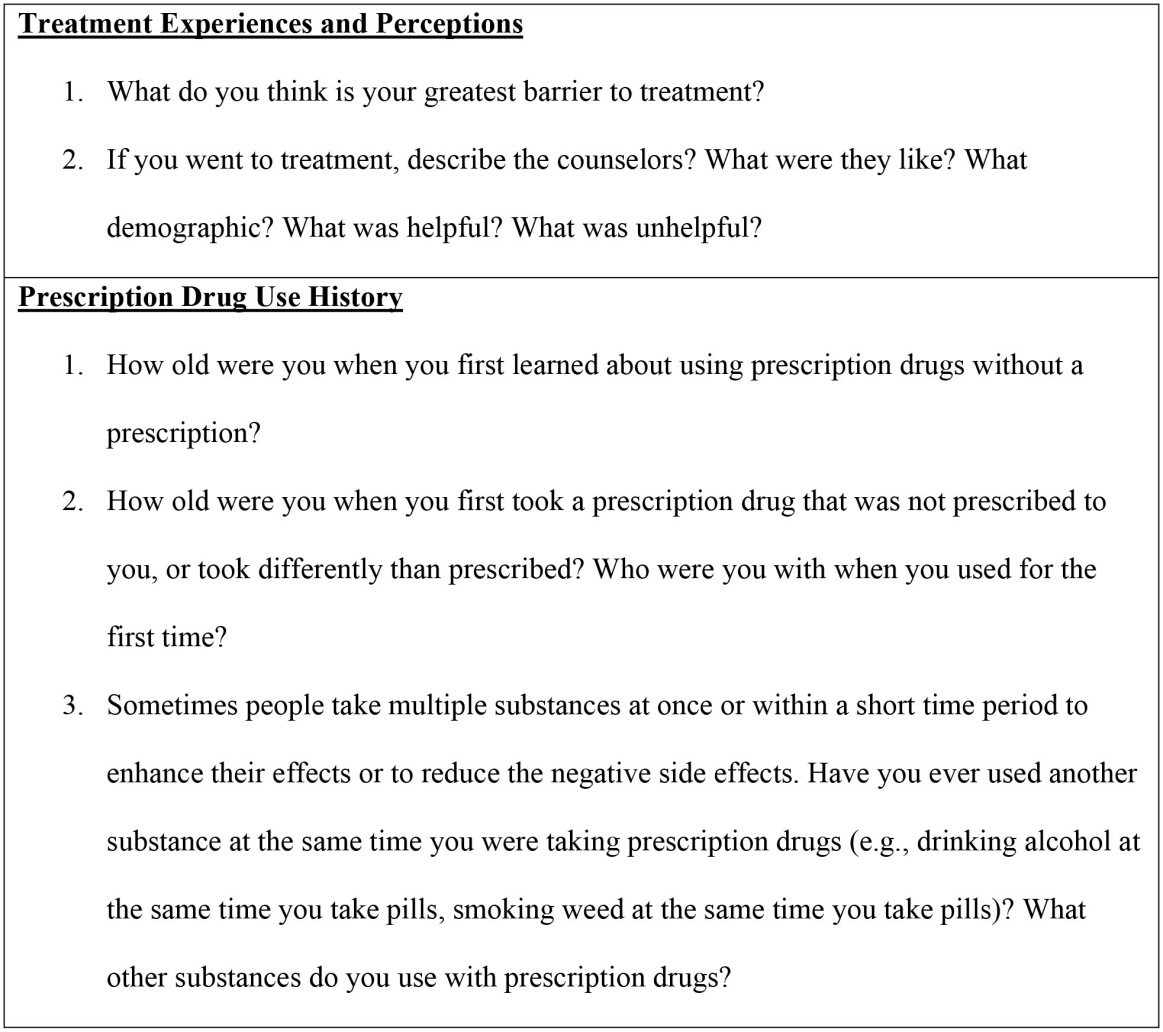
Sample questions: Aim 2 interview portion.

Computer Assisted Personal Interviewing (CAPI) software and Audio Computer Assisted Self-Interviewing (ACASI) software from the Questionnaire Development System (QDS™) are programmed on project laptops for Aim 2 data collection. Data is collected in interviews led by African American research staff trained to work with participants concerning the sensitive topics discussed in both the CAPI and ACASI study questions. CAPI study questions are administered using an open-ended interview at the beginning of the participant's data collection session. These questions gain insight into the participants' perceptions of drug treatment and their history with opioid misuse. Sample questions are provided in Figure 3. ACASI questions are administered using an electronic survey. The survey reads each question to the participant and provides a set of answer options. Participants click on their desired response independently or with help from research staff if they are not comfortable with the technology. Sample measures are provided in Table 1.

**Table 1:** Sample measures: Aim 2 quantitative.

	**Social**	**Personal**	**Demographics**
Factors assessed	• Social Support • Family and Peer Relationships • Perceived Social Norms	• Active Coping • Religiosity/Spirituality • Ethnic Identity • Distrust in the Healthcare System	• Employment • Income • Housing Status • Healthcare Access • Criminal Justice Exposures
Sample instruments	- Addiction Severity Index-Version 6 (ASI-VI) (39) - Perceived Norms of NMPOU and NMPDU (40)	[-]Multigroup Ethnic Identity Measure (MEIM) (41, 42)	Addiction Severity Index-Version 6 (ASI-VI) (39)

Consent for the current project is obtained prior to beginning study activities. Participants may decline participation or leave the study at any time. All participant information is de-identified with a participant ID number and can only be identified by some members of the project team. Participants of Aim 2 earn $40 for completing study activities and an additional $10 for completing an optional urine drug screening. Aim 2 participation consists of one 90–120-min computer-led interview administered through ACASI, and an open-ended interview conducted by the study's staff. After study activities are completed, the participants of this aim are released from study activities.

##### Analytic plan

When all data from Aim 2 has been collected, it will be entered into a statistical program to create paths and mediation analyses. These analyses will help to assess relationships between NMPOU, illicit drug use, and potential mediators and moderators of NMPOU, illicit drug use, and the use of treatment services. An example of hypothesis mediation pathways is examining the relationship between opioid use, treatment completion, and existential wellbeing. Standardized regression estimates and *p*-values will be provided with diagrams. Using the full dataset, latent class analyses will be conducted to identify groupings of non-medical opioid users. Creating these groupings can be beneficial in future interventions by identifying individuals who may be at higher risk or benefit from intervention.

#### Aim 3

##### Expected outcomes

Study activities for Aim 3 are currently in progress. Aim 3 data is expected to lay the groundwork for treatment intervention among African Americans who engage in non-medical opioid use. This is expected to be achieved by identifying patterns from Aim 1 and Aim 2 to create messaging and gain insight on these messages from participants. Data for this aim can produce papers catering to generational and biological sex differences in intervention attitudes.

##### Participants

Identical to Aim 1, Aim 3 requires 40 (*n* = 40) participants, five biological males and five biological females, from each age cohort. Five participants in each sex and age cohort allow for different lived experiences to be reflected in data analysis. The informational power is also identical to the first aim. Eligibility criteria are identical to Aim 2. Participants must be willing to be audio-recorded at the time of their interview. Participants cannot have previously participated in Aims 1 or 2 due to concerns with biased responding.

##### Data collection, incentive, and retention

Like Aim 1, participants engage in a 60- to 90-min face-to-face interview. Interview sessions instruct participants to “think aloud” as they read messages on a computer screen. After, participants rate how much they liked the message and its relevance to substance use treatment participation, opioid use, and other relevant factors such as aspects that may help an individual through treatment. Participants then state which messages they prefer and their reasoning. Behavior observations, notes of questions asked by participants, and how they responded to each question is recorded by the interviewer. Participants also rate messages on different dimensions and from different sources to evaluate their relevance. Sample messaging for this aim and its corresponding interview questions can be found in Tables 2, 3. Like in Aim 1, in-depth interviews will continue until saturation for each biological sex/age group is reached (*n* = 5) and 40 total interviews are conducted. A trained African American research staff member audio-records and transcribes the study session with integrated field notes of behavior observations. Similar to Aim 1, a member of the research staff reads each question to the participant in the same words as they appear on the interview script to reduce responding bias. Aim 3 participants can earn $40 for the completion of aim-specific activities. In Aim 3, participation in the study will end after the study activities are completed interview.

**Table 2:** Aim 3 messages categorized by generational cohort.

**1955–1969**	**1970–1979**	**1980–1994**	**1995–2007**
What if you could learn how to be the life of the party without drugs	We can't always cope with drugs. It becomes a cycle. You're the only one who can break it	Kids can tell when you have had your “medicine”	If your anxiety is driving you to pills, make sure it's medications made to treat anxiety, not opioids
You look out for your people – what if your support looked out for you, too?	I know you can quit when you want… but sometimes your body on opioids won't let you	Your children deserve a version of yourself that has overcome addiction	Imagine somebody who can't even afford labels trying to label you. Hilarious. Do what you need to do to kick your drug use
Overdose and addiction can occur even if you don't use “that much”	Similar to death. Overdose don't discriminate	You won't know if you took a bad pill but your grieving family will	When you know better you use better… or something like that

Some messages were applied to more than one cohort based on their potential relevance.

**Table 3:** Sample interview questions for Aim 3.

**Question category**	**Interview questions**
Effectiveness rating	Please rate this message on a scale of 1 to 3 based on the effectiveness of (e.g., increasing overdose prevention/increasing cues to treatment/reducing drug use)
Reactions to the messaging (Appeal)	What did you like the most about the messages?
Reactions to the messaging (Clarity)	Tell me in your own words what you think the main point of the message is?
Reactions to the messaging (Accountability)	How would you change this message to motivate you to do something different?
Closing questions	What would be your reaction to reading this message in your daily life?

##### Analytic plan

Aim 3 analyses will focus on identifying and summarizing patterns of experiences related to the content and reactions of the messages. Data will be analyzed using thematic framework analysis (35, 36) involving a senior scientist who is an expert in qualitative research, a trained research assistant, and another senior scientist on the project. After being familiar with the content of the study, a similar analytic process to Aim 1 will take place. The six phases of this process are as follows:

1. Data was read multiple times to pull memos highlighting aspects of interviews that are relevant.
2. Data was coded and organized into meaningful groups based on its elements.
3. Coded data was re-read to connect codes into themes. A thematic map represented the relationships between the two.
4. Themes were re-evaluated to consider codes not yet fitting into the existing themes. Adjustments to the thematic map took place in this stage in this stage.
5. To ensure themes highlight participants' intended meaning and relate to the purpose of the study, two participants were chosen at random from each age and biological sex cohort (*n* = 16) to review if their experiences are accurately represented by the themes selected. An additional incentive was offered to these participants.
6. Recurring themes were reviewed with the entire research team and analyzed the key findings. These discussions helped finalize the questions used in Aim 2 of the study.

Memos, codes, and themes will be developed to correspond to the meaning of participant responses. Codes and themes will be analyzed again to ensure each response is connected to the larger meaning of the dataset. ≥90% reliability will be acceptable after each coding of a subset is checked.

### Resources after the interview

After the participants completed study activities, they were offered resources for housing, food, clothing, and mental health and substance use treatment options in the area they were interviewed. These resources are provided after their completion to promote the next steps in their rehabilitation. Another resource given to participants is naloxone, an opioid reversal drug administered intranasally. The research staff member who administered the interview also describes how naloxone is used and teaches participants how to use it. Participants are also given a handout with their naloxone to remind them of how to use and store the drug.

## Discussion

The REFOCUS study examines the complex, generational, and biological sex factors and patterns of opioid misuse among African Americans. These factors have not yet been considered when examining the opioid epidemic within African American communities. By understanding these factors, treatment approaches can be initiated to be more suitable for African Americans who misuse opioids and ultimately decrease overdose deaths. Different aims of the study bring different barriers (e.g., recruitment demands, duration of data collection interviews) to study completion. However, the strengths of this study were strategically implemented to decrease the negative effects of the study's limitations.

One limitation of the study is the large sample needed to complete Aim 2 according to the power analysis (*n* = 800). When obtaining large sample sizes in research, there can be changes in the rates of participant enrollment throughout the project. However, the study's focus on innovative recruitment techniques has been successful in recruiting a large data sample. With these efforts, we have collected about 96% of the needed participants in Aim 2 (*n* = 766). Beyond posting flyers and the referral process, African American research staff recruited participants by connecting with individuals in neighborhoods and the community to discuss the importance of the research study.

Research staff also partnered with local organizations that worked with African Americans in the community to reach potential participants and educate them about the importance of the REFOCUS study. Community partners include but aren't limited to, substance use treatment facilities, food pantries, nail technicians, lash technicians, mental health initiatives, community centers, and employment centers. Specific age and biological sex cohorts were targeted based on perceived activities members of these groups would attend. For example, research staff went to local bars and clubs to recruit members of the youngest cohort and bingo halls to recruit the older cohorts. For biological sex-specific recruitment, the research staff attended events catered to biological sexes, such as male and female health fairs and biological sex-specific housing shelters. A limitation to such recruitment techniques is the limited regional representation of participants completing the study. This may impact how the study's findings are generalized to African Americans in other regions in the United States.

The REFOCUS study works with participants with potential intersecting characteristics that may impact their ability to complete the study. This can pose a limitation for the study that impacts data collection accuracy and its ability to meet participant requirements. Another strength of the REFOCUS study is that standard operating procedures ensure an accessible, accommodating environment for every participant. The in-person screening includes questions from the Mini-Mental State Examination (MMSE). The participants' responses signify their level of orientation and ability to engage in the interview. Also, trained African American research staff members conduct the MMSE. By utilizing African American research staff, the study increases the likelihood of trust and rapport building with participants. Thus, participants may be more comfortable sharing their honest answers.

Another way the study provided an accommodating environment is by providing refreshments, allowing participants to take breaks when requested, and using different techniques to keep participants focused on the research interview. If these methods do not work, clinicians reschedule their appointment for a different time to ensure data quality and keep the data previously collected to the point of termination of the initial interview. By addressing potential barriers with the study's strengths, the study anticipates collecting quality data on the experiences of African Americans who misuse opioids. Collecting quality data increases the study's validity. Also, it allows for substance use interventions to incorporate biological sex and generational values important to African Americans, which may help decrease opioid overdose rates.
